# End-of-life doulas: international reflections on a transnational movement

**DOI:** 10.1177/26323524231186826

**Published:** 2023-07-25

**Authors:** Marian Krawczyk, Emma Clare, Erin Collins, Sarah Farr, Elizabeth Johnson, Jennifer Mallmes, Annetta Mallon, Kelly Oberle, Jennifer Rigal

**Affiliations:** College of Social Sciences, University of Glasgow, Rutherford/McCowan Building, Crichton University Campus, Dumfries, Scotland DG1 4ZL, UK; University of Derby, Derby, UK; The Peaceful Presence Project, Bend, OR, USA; College of Social Sciences, University of Glasgow, Glasgow, UK; The Peaceful Presence Project, Bend, OR, USA; Douglas College, New Westminster, BC, Canada; Western Sydney University, Penrith, NSW, Australia; College of Social Sciences, University of Glasgow, Glasgow, UK; College of Social Sciences, University of Glasgow, Glasgow, UK

**Keywords:** death doula, end-of-life care, end-of-life doula, EOLD

## Abstract

This review article summarizes the findings from the first virtual International End-of-Life Doula Symposium, held over 3 days on 25–27 April 2022. More than 40 people attended from seven countries, predominantly from Australia, Canada, the United States and the United Kingdom, and they were primarily experienced practitioners. In this article, we focus on participants’ topics of conversations and experiences that were relevant across international boundaries, organized through the symposium themes of developments, disruptions, dilemmas and directions. All authors took de-identified handwritten notes across the 3 days of discussion, as well as reflexive notes about our own thoughts and perspectives on the topics discussed. We then collated our notes and abductively focussed our analysis on topics that generated significant conversation and/or came up repeatedly within the overall symposium themes, as well as trying to capture any unexpected issues and perspectives. We identify and summarize a wide range of interests and concerns within the development of the end-of-life doula (EOLD) role. We provide a model for integration pathways within existing health care systems, as well as an innovative conceptual framework synthesizing key intersecting developmental issues that are relevant across regional and national boundaries. The symposium was the first opportunity for EOLDs to collectively discuss their work and interests within an international context. Our findings indicate that there are fundamentally similar developmental issues across countries, along with some variations. As the first international event of its kind, our ‘state of the field’ summary review of the symposium holds significant insights relevant to both national and international contexts, and to a diversity of stakeholders interested in the development of this new care role and emerging transnational movement.

## Introduction/background

End-of-life care has evolved significantly with the ongoing expansion of palliative and hospice care. Concerns for person-centred, equitable and compassionate end-of-life care have increased, resulting in changes in care practices and priorities. These concerns have arisen in tandem with globally ageing populations, economic constraints for health and social services, and a growing interest in community-led care. As part of this changing landscape, a new care role is emerging that may hold the potential to define a new paradigm in end-of-life care. While there is no mutually agreed descriptor of this role, the appellation ‘end-of-life doula’ (EOLD) is commonly used as an umbrella term to identify lay people (primarily women) who provide nonmedical supports, informed companionship and resources for people with a serious life-limiting illness or who are nearing the end of life, including those close to them. These supports can include social, emotional, practical and spiritual elements before, during and after death, depending on need and on an individual EOLD’s skills. Interest is developing as to how EOLDs may be able to support community-based and community-led end-of-life care and advocates propose that they also hold a larger potential to reconfigure the culture and care practices in dying, death and bereavement.

There is a small, but developing, body of literature and research on EOLDs, with work detailing the EOLD role and its potential benefits,^1,2^ the diversity of practices within this unregulated field,^3^ as a new model of care,^4,5^ and some historical and contemporary antecedents.^6–8^ However, there is little research that explores this new care role across international boundaries. To address this gap, this article reports the findings from the first virtual International End-of-Life Doula (EOLD 2022) Symposium, held over 3 days on 25–27 April 2022.

## The EOLD symposium

The EOLD2022 symposium emphasized direct engagement and collaboration with practitioners in creating a shared space to (1) collectively share and reflect on individual practices; (2) explore overlap and differences in regional/national practices and concerns and (3) map future research interests across an international landscape. The symposium was led by the first author, in co-production with all the authors who come from the four countries where EOLDs are most active: Australia, Canada, the United States and the United Kingdom. It was the first international symposium or conference of its kind.

In this article, we summarize participants’ topics of conversation and experiences that were relevant across international boundaries, organized through the symposium themes of developments, disruptions, dilemmas and directions.

- The ‘developments’ theme focussed on the roots of the field of practice and what we can learn from the emergence of the contemporary EOLD role.
- The ‘disruptions’ theme considered EOLDs as cultural ‘change agents’ with the capacity to engender disruptive innovations in end-of-life care.
- The ‘dilemmas’ theme relates to the many issues and challenges facing current practitioners as the field expands.
- The ‘directions’ theme related to the possible future directions for EOLDs, both as individuals and collectively.

These themes were offered as provocations, based on previous research and practitioner consultation. The intent was to explore these ideas and concerns from multiple positions. We use participants’ quotes throughout to illustrate the key topics which we identified within each symposium theme.

The symposium was an ‘invitation-only’ event to ensure that it primarily involved those who were experienced practitioners. Potential participants were identified through the authors’ extensive professional and research networks. We also asked well-known practitioners who they would recommend inviting and invited those people as well. In a few instances, we invited people who were important to the development of the field through their position in academia, health care and/or government. Potential participants (*n* = 101) were sent personal invitations 5 weeks before the event. Seventy-nine participants registered. This resulted in a﻿ diversity of practitioners who provide a wide range of services, support and care focussed on decline, dying and death, including post-death activities. Many symposium participants did not personally identify as an end-of-life doula. The authors have chosen to use this descriptor due to its international recognition but we also recognize this is a topic of vigorous debate and it is discussed in some detail later in this article.

To address the different time zones, the virtual symposium format included pre-recorded talks followed by live Q&A (Day 1); unstructured Zoom ‘drop in’ sessions to connect and talk with other participants (Day 2) and small, facilitated group Zoom sessions focussed on specific questions taken from the previous day’s discussions, along with a closing panel discussion reflecting on the main themes, ideas and issues that arose over the 3 days (Day 3). There were varying numbers of participants during symposium presentations, with an overall average of 30 participants per session, with 44 participants overall attending at least one session. Countries represented across the sessions include Canada, the United Kingdom and the United States, Australia, Spain, Sweden and Columbia. The over-representation of participants from global north countries reflects the geographic pattern of growth of this field of practice^3,7^ and awareness of this was a significant point of discussion within the symposium. We did not collect individual characteristics of participants. While the symposium sought out a range of different speakers, of all our seven speakers identified as female. Two were women of colour, and one identified as Metis. It is not known how many symposium participants identified as other than white, however, there were only a handful of participants who did not appear to be visibly from a white ethnic background. There were three men among participants.

Each project collaborator (AM, EC1, EC2, EJ, JM, EC2 and MK) attended four planning meetings, recorded a 20- to 25-minute talk based on a symposium theme, participated in live Q&A and the closing panel discussion, and facilitated small group conversations during the symposium. Volunteers also attended these meetings and were instrumental to the success of the symposium, and were either practitioners (SF) or training to become EOLDs (KO and JR). All authors took de-identified handwritten notes, at times verbatim, across the 3 days of discussion, as well as reflexive notes about our own thoughts and perspectives on the topics discussed. We also took note of comments within the online chat function. We then collated all our notes and abductively focussed our analysis on topics that generated significant conversation and/or came up repeatedly within the framing of the overall symposium themes, as well as trying to capture any unexpected issues and perspectives.^9^ While we were not seeking to generate new theoretical frameworks, this approach enabled us to develop conceptual innovations regarding the central issues within the development of the EOLD movement internationally (i.e. Figure 2). All authors contributed equally to the development of this article, meeting twice after the symposium to discuss and organize the main points within each theme, and commenting on and editing the initial drafts. While we have sought to minimize overlap, there are some topics of discussion which cross themes. In these instances, we frame our discussion through its relationship to the specific theme. We offer a summary of all the main discussion topics and key concerns within the symposium themes in Table 1. A more modest and earlier summary of symposium proceedings is available as an e-print,^10^ and this article contributes substantially new context, discussion and connections to relevant literature.

**Table 1.** Main topics of discussions and key concerns within symposium themes.

Symposium themes	Main discussion topics and key concerns
Developments	• Loss of experiential knowledge and community supports in dying and death • Close relationship between pregnancy/birth and dying/death • Caregiving historically understood as ‘woman’s work’ • Growth of contemporary hospice and palliative care
Disruptions	• Unique philosophy, knowledge and skills • Enhancing death literacy • Involvement with compassionate community initiatives • Practicing cultural humility • Addressing structural inequalities Championing inclusionary practices
Dilemmas	• Certification, regulation and/or professionalization • Valuing different forms of knowledge • Consequences of naming • Payment as empowerment and as problem • Integration with formal health care systems and compassionate community initiatives • Relationship to hospice and palliative care • Lack of practitioner diversity
Directions	• Responding to ongoing changes in contemporary dying • Retaining meaningful ‘vanguard’ role as cultural change agents • Differing integration pathways • Further training and practitioner support • Need to balance development tensions • Need for research and international discussions

## Main themes

### Developments

This theme relates to the history and emergence of the contemporary EOLD role and associated practices. The focus is on considering how development of the EOLD role is situated within larger social, economic, political and cultural changes in the global north. For participants, reflecting on the diverse developmental roots of the EOLD role and field of practice also helped to contextualize how various historical antecedents might be shaping current practices and challenges, as well as potential future practice pathways. Symposium participants highlighted four main aspects when discussing the development of EOLDs: (1) the historic loss of experiential knowledge and community supports in dying and death; (2) the close relationship between pregnancy/birth and dying/death; (3) how caregiving historically has been seen as ‘women’s work’ and (4) the impact of contemporary hospice and palliative care.

By far, the most common observation about the development of the contemporary EOLD role was in relation to the historic loss of individual, family and community-based knowledge in dying, death and bereavement.

The end of life doula role is not really new; it has existed for always, in all ways, at the community level. It was the negative impact of the medical system that has driven social death-caring into the medical arena and disempowered people to care for their own . . . it’s connected to and emerging as a response to the medicalization of birth and death.

Many practitioners saw themselves as ‘reclaiming’ these informal lay practices and there was a strong sense of loss expressed for these forms of community relations, knowledge and rituals. Similar to the above participant, others also commonly referenced the close relationship between pregnancy/birth and dying/death in discussing the development of EOLDs. This connection was invoked through highlighting how the term ‘doula’ originated with birth doulas emerging in the 1970s as part of the natural birth movement in the United States, in linking the beginnings and endings of life as transitional liminal times requiring special care and attention, and/or through articulating both as life processes that have become overmedicalized events subject to specialist expertise (Not all EOLD agree with the aetiology of this term (for further discussion, see Krawczyk & Rush, 2020).

The discussions about the development of the EOLD movement were also interwoven with reference to the ways caregiving has historically been seen as ‘women’s work’. This work was understood to be particularly important during birth and death in previous eras, where laywomen with experiential knowledge and skills provided most of the care. Symposium participants privileged this historically gendered and community-based labour as something incredibly valuable yet currently devalued and made invisible within contemporary health care systems. The concern for, and close mapping of, the EOLD role to historical community ties and the importance of women’s experiential knowledge and labour at the beginnings and endings of life has been noted elsewhere.^6,7^

A somewhat unexpected yet significant point of discussion centred on the rise of EOLDs within the era of hospice and palliative care. Some symposium participants felt that the development and mainstreaming of hospice and palliative care has signalled a cultural change in valuing and addressing the needs of those with life-limiting illnesses and their social networks, which in turn has opened space for EOLDs. Others took a somewhat opposing stance, arguing that it has been the inability of hospice and palliative services to meaningfully engage in holistic care ‘at scale’, despite its original mission,^11^ which has ironically increased the need for EOLDs.

### Disruptions

This theme considers how EOLDs articulate an identity of ‘doing things differently’ with the capacity to beneficially disrupt existing health systems and cultural norms in providing innovative end-of-life care. The symposium conversations revealed the central importance which practitioners placed on activities that radicalize dying, death and bereavement. This has also been articulated elsewhere with EOLDs identifying as ‘cultural change agents’ who are reconfiguring relations of care in ways that facilitate a collective shift in dying, death and bereavement, which necessarily includes but surpasses the individual ‘good death’.^3^

Symposium participants highlighted multiple practice pathways to positive disruption, including: (1) a unique philosophy blended with specialist knowledge and skills; (2) public advocacy about the benefits of the EOLD role; (3) practices for enhancing death literacy; (4) practicing cultural humility across all cultural contexts, including one’s own; (5) addressing structural inequalities in end-of-life care and (6) championing inclusionary practices and practitioners.

The most common reference employed by symposium participants in marking their distinction from other care roles was a unique combination of philosophy, knowledge and skills. This was framed as EOLDs offering flexible, informed and compassionate supports, which ideally leads to a heightened capacity and overall sense of wellbeing, both for the person nearing the end of their life and those close to them. Determining and advocating for the person’s and family’s needs and wishes, educating people about what to expect and the benefits of advance care planning were all specifically referenced and seen as key to ‘dying well’. Along with navigating the bureaucratic aspects of dying, there was also significant weight given to the idea that the EOLD role blends both ancient knowledge and modern skills, with the disruptive potential to draw from and equally value both, contrary to contemporary biomedical care practices.

Symposium participants also talked about their public advocacy activities which are designed to increase knowledge about what EOLDs do, including how their services differ from existing services, and the potentially unique benefits of their care. Micro- and meso-level benefits encompassed individuals, their social networks and the larger communities these people are situated within. Macro-level benefits were also linked to simultaneously supporting and disrupting larger organizational systems, where EOLDs can address gaps in health services and/or integrate within interdisciplinary care teams while also challenging the current norms within end-of-life care infrastructures, such as the conventional funeral industry. Specific community-based advocacy activities included media work, offering free community or virtual information sessions, as well as social or educational events, such as facilitating Death Café events and advance care planning sessions. Participants also discussed more informal, everyday forms of advocacy:

The name ‘doula’ itself lends itself to conversations about dying and what the doula role is, and this makes us change agents by opening up dialogue right from the beginning.

These socio-educational activities were commonly articulated as a way through which EOLDs act as cultural change agents to enhance death literacy and consequently death competency. Death literacy is defined as ‘the knowledge and skills that people need to make it possible to gain access to, understand, and make informed choices about end-of-life care and death options ’ (p. iv),^12^ and death competency as ‘a range of human skills and capabilities in dealing with death, as well as our beliefs and attitudes about these capabilities’ (p. 161).^13^ Within symposium conversations, an important component of enhancing death literacy and competency was through EOLDs assisting communities to ‘re-member’ their collective capacity to care for their dying and their dead. Many participants stated this as a key goal of their work through ‘facilitating more open conversations about dying and death within a death-avoidant society’, acknowledging that this requires a ‘disruption’ of normative conventions.

This perspective dovetailed with discussion about the place of EOLDs within Compassionate Community initiatives in many countries. There was considerable discussion about the possibilities offered to EOLDs for changing the ways in which dying, death and bereavement are currently navigated through participating in and/or leading Compassionate Community initiatives. Our American collaborators highlighted possible roles of EOLDs within the Compassionate Communities’ model of care. This included as volunteer ‘companions’ who refer to more complex levels of care and support as needed; as referral resources for health care providers and health systems and as knowledgeable, resourced individuals within a community who share information and skills within their other roles in the community.

Discussions about disruptions also reflected the need for continual awareness of one’s own socio-cultural position and resulting beliefs and values. The concept of cultural humility was referenced throughout the symposium, initiated by a collaborator’s (JM) presentation on Day 1, where she offered the Mi’kmaw ‘two-eyed seeing approach’^14^ to differentiate between cultural competence and cultural humility in discussing Indigenous end-of-life issues in Canada. The approach is an integration model, whereby pathways to inquiry and solutions require a perspective, which combine both Indigenous and Western ‘lenses’ – two different ways of ‘seeing’ that make visible the histories of *both* ways of seeing. This differs from an uncritical use of ‘cultural competence’, which can de-historicize knowledge, has potential to stereotype cultural groups and often assumes standardized communication techniques.^15,16^

During the symposium, there were two overlapping ways that participants employed cultural humility. First was the understanding that as individual practitioners, they may not always be able to meaningfully address specific cultural issues that shape the end of life. Second was the critical self-reflection as to how EOLDs can disrupt inequitable systems and move towards greater inclusion and equity. Practicing both forms of cultural humility were seen as radical acts within existing social systems, and for many an important part of the EOLD role. Correspondingly, many symposium participants were deeply concerned with structural inequalities shaping the end of life. Canadian and Australian participants highlighted institutional racism and the legacy of colonialism on Indigenous peoples, particularly as it related to traditional knowledge, intergenerational trauma and geographic dislocation at the end of life.^17^ American participants added concerns about the needs of Black and Latino people at the end of life that often go unaddressed within care models founded on White middle-class experiences and values.^18^

Symposium participants from all the countries represented discussed health care as an equity issue and identified the need to address socioeconomic disparities at the end of life, including the need to offer free or sliding scale services. Some participants were concerned that integrating EOLDs into existing health systems will not necessarily result in increased access for people experiencing structural inequalities, given that health systems can exclude and even render some people invisible. While not all symposium participants expressed being motivated by issues of social justice and delivery of equitable services, there was a relatively unified position that EOLDs need to support inclusionary practices and practitioners.

### Dilemmas

There were many engaged conversations about current and future challenges and dilemmas in the field of practice. The main issues were: (1) concerns for standardization, (2) the relationship between ‘lay’ experiential knowledge and ‘codified’ professionalized knowledge, (3) the consequences of naming the role, (4) payment as empowerment and as problem, (5) integration within formal health care systems, (6) the relationship to hospice and palliative care and (7) lack of practitioner diversity.

The challenges of certification, standardization and/or professionalization of EOLDs are well-known, as are the diverse and potentially polarizing views that various practitioners hold about the nature and scope of the role.^3,19^ This heterogeneity within the field was also reflected in the range of views shared during the symposium. In relation to certification, concerns were expressed about the variable quality of programmes where people could call themselves ‘certified’ with minimal or poor quality training (or even no training at all). Some participants framed the need for standardization as ‘quality control’ and to ensure a regulated ‘scope of practice’, and/or ‘core competencies’ across certifications. In relation to professionalization, proponents referenced different existing care models (e.g. personal care workers, social workers) which may enable EOLDs to be integrated within health care and ‘renumerated appropriately’. While some practitioners expressed strong feelings against any form of standardization, many symposium participants held these views in tension – seeing the potential for both beneficial and problematic consequences.

Regulation will give credibility and visibility – and it can limit and take away power from those who ‘can’t play game’, [such as being able to] afford licensure or regulation fees, etc.

Overall, there was significant interest in trying to figure out a ‘middle’ path in developing the EOLD role, focussed on ‘flexibility’ and welcoming both practitioners drawn towards standardization and those wishing to remain independent.

Concerns about certification, standardization and professionalization were closely entwined with concerns about valuing different forms of knowledge. One of the key benefits participants articulated about EOLDs is their capacity to combine two desirable forms of knowledge. The first is a ‘lay’ embodied knowledge which only emerges from the lived experience of accompanying the dying and is in part a historical continuation of ‘ancient’ or ‘traditional’ knowledge. Others described this tacit knowledge as an intangible emotional or affective perspective, framing it as ‘heart work’. The second type of knowledge is an ‘expert’ professionalized knowledge about contemporary health and bureaucratic infrastructures which structure dying and death in the 21st century. This explicit form of knowledge can be easily codified: written down, standardized and evaluated. Symposium participants were clear that these two forms of knowledge do not always co-exist seamlessly, and not all participants were interested in being experts in both forms of knowledge. Several participants further questioned how these forms of knowledge could be conjoined without diminishment:

How do you make the foundation of your work a marriage of the experiential with practical wisdom with knowledge of system realities?

There was widespread agreement that naming the role was important, although there was a range of positions as to whether the name should be standardized. While the term EOLD has significant popularity, it was not a desirable descriptor for many participants, with reasons including but not limited to its cultural specificity, little public knowledge of the word ‘doula’ and questioning the relations of power which require standardization of names and practices. An informal poll during the symposium about the role descriptors people used produced: EOLD, end-of-life companion, end-of-life guide, death doula, death midwife, soul midwife, companion guide, therapeutic companion and death advocate, as well as single word descriptors, such as consultant, advocate, guide, companion and navigator. As explored in other studies, this naming variation was further complicated by some participants sharing that they use different descriptors in different contexts depending on who they are talking to or working with.^3^ Overall, participants expressed that the way the EOLD role is promoted is inconsistent and complex, which in turn impedes public awareness and uptake. However, while there was general agreement that a mutual descriptor or umbrella term would help with ‘cohesiveness in the field’, ideally practitioners also wanted it to be inclusive and broad enough not to exclude a diversity of practitioners. Several participants also highlighted the challenge of standardizing a role descriptor without a clear scope of practice or standardized competencies.

Another prevalent and lively dilemma surrounded the issue of payment, with much discussion and debate about framing these practices as ‘work’. Some participants connected the issue of payment to the historic relationship between emotion and care work, volunteering and gender.

It’s an important point too about ‘heart work’ often being applied to roles which women traditionally work in, and in volunteering, and how this is very gendered.

This was contrasted to the perception of health care more generally as non-gendered. While all forms of volunteering – particularly hospice – were seen as an important source of experience for those new to the field (whether volunteering in the EOLD role or not) and a desirable form of community-building, volunteering was also seen as having the potential to devalue EOLD’s skills and services. Others also argued that expecting EOLDs to provide their services without a fee limits who can ‘afford’ to do this work. For one particularly emphatic participant, providing any voluntary EOLD services was seen as potentially furthering gender inequality:

My perspective is that women do enough unpaid labour. Volunteering devalues the field, we spend time, money and energy training and upskilling which has value, and being paid means that there is the opportunity for equity-based service provision when someone in need presents themselves.

Participants discussed different payment models as part of this concern. Some shared how they charge for their services, and which included sliding scales or pro bono work, and/or belonging to organizations or practice collectives that have (small) budgets to subsidize people who cannot afford services, funded in part by self-donated fees from practice, training, donations and public talks. A few discussed their work primarily as volunteer-based or as a non-profit endeavour, the latter including organizations that may include both paid and volunteer practitioners. Many participants expressed an interest in working towards having EOLDs financed through state-provided universal primary care, funded at the point of access and thereby accessible by all. Others considered how their services might be reimbursable (within private or mixed health insurance systems), and how this might work particularly well in regional health systems that offer personal health budgets. Interestingly, a UK participant stated surprise that other country participants used the term ‘clients’ since she felt this is a more contested term in the United Kingdom than other terms, such as ‘people we support’. In addition, one South American participant noted that many people in her country have no universal health care or health insurance and that ‘people just organize themselves and get on with it’ rather than try to secure formal financing at states or systems-level. The issue of payment was also referenced in relation to Compassionate Community initiatives. Symposium participants were enthusiastic about their involvement in supporting communities to become more death literate, but some were also uncertain as to the role of EOLDs within these initiatives. In the words of one participant, EOLDs could be seen as ‘creating billable clients from community members’. This and other potential challenges of integrating EOLD practice within the Compassionate Community-led frameworks for action have been considered elsewhere.^20^

Participants across all the countries displayed reflexivity regarding the tension between the potential of EOLDs to further commodify end-of-life and post-death care with the desire to receive remuneration for their work, including ‘being able to make a living’. Rather than necessarily seeking a consensus, most participants expressed appreciation for open discussion about how to equitably develop the role and services in the context of the increasing entrepreneurialism surrounding dying, death and bereavement within late capitalism and mixed economies of care.^8,21^ There was widespread (but not universal) agreement among participants about the benefits of a blended model whereby EOLDs could choose whether to offer their services for a fixed fee, through subsidy, a sliding scale and/or for free.

A dilemma that involved but surpassed issues of payment was the concern for integrating within formal health care systems, including the relationship to hospice and palliative care. As mentioned previously, some participants felt that hospice and palliative care had laid the foundations for their own role and development. This included hospice programmes beginning to take interest in and/or employing or referring to EOLDs. Other participants shared how they worked alongside and supported hospice and palliative care teams. However, not everyone wished to align themselves. The mainstreaming of palliative and hospice care in the global north was seen by some as generating the need for EOLDs in the first place, as health care system constraints have compromised the whole-person care once promised in the early years of its development. For example, there were several cautionary stories shared about the development of American hospice care once it became a reimbursable service under Medicare and how, in many instances, providing this form of care is now in name only as it has been consumed by large investors.^22,23^

There were also discussions about the benefits of working directly for individuals and families rather than a health authority or other health care institutions. For example, some participants shared stories about their ability to continue home visits during the first waves of the pandemic, where other health care providers could not (due to regulations or acuity of workload). At the same time, there was a realization, in the words of one participant, that ‘it doesn’t benefit those we care for to work only in an alternative, “outside of the system”, way’. Other participants spoke about their ambivalence between seeking legitimacy and some form of health system integration with the desire to disrupt existing ways of doing things that might be best achieved by being outside of those systems.

Finally, frustration was expressed that even as the EOLD movement offers potential disruption and innovations in end-of-life care, it is also reflecting and reproducing a narrow range of perspectives and needs:

There’s a lot of discussion about [how] diversity and inclusion is at the heart of EOLD organisations and practice. Whilst we strive for this in practice, the reality is that the vast majority of our members and practising EOLDs are white, middle-class women.

This awareness was connected to EOLD’s capacity to be meaningful social change agents. Representational dilemmas included how privilege shapes the ability to practice, peoples’ access to their care, and in generating assumptions about the types of care needs for people who do not have White middle-class norms, experiences or resources. This was particularly highlighted by speakers Jennifer Owens and Valoria Walker who each provided examples of how mainstream medical services may ignore, or even be openly antagonistic to, the needs of Black Americans and/or women of colour. Furthermore, several participants expressed concern that EOLDs may be using cultural death practices that have been stolen or lost through processes of colonialization, potentially causing further disenfranchisement. There was also some discussion of the need to be aware of ‘ableist’ language that may be unreflexively used by some practitioners, such as ‘walking’ alongside someone.

### Directions

Amid such rich conversations, there were a range of topics discussed regarding the potential directions and future of the field. These included: (1) addressing changes in contemporary dying; (2) ensuring a meaningful vanguard role as cultural change agents; (3) differing integration pathway within health care systems; (4) further training and practitioner support; (5) balancing/negotiating developmental tensions and (6) the need for research and international forums.

Discussions about how to respond to changes to dying, death and bereavement in the 21st century included extending EOLD care to sudden death, both unexpected and expected; to address the rise of ‘ambiguous dying’ in old age, including multimorbidity, dementia and frailty;^24^ and increased interest in designing end of life and post-death activities, such as ‘going away parties’ and ensuring an ‘environmentally friendly’ death. Participants from all countries also expressed a particularly strong interest in discussing how to meet the needs of people who wish for a hastened death. The focus was not only on the needs of the individual requesting assisted dying or those who voluntarily stop eating and drinking (VSED), but also those who are close to them.

As part of their reflection about these cultural, demographic and epidemiological changes, participants expressed that their future role requires being at the forefront of responding to these changes. This continued vanguard role as ‘cultural change agents’ included understanding that end-of-life care is a political act (or at a minimum is situated within political contexts) requiring a social justice and equity approach to dying, death and bereavement. At the micro-level, this was often articulated as needing to ‘empower’ individuals and their families through, in the words of one participant, ‘self-determined palliative care; what care you want when you want it’. EOLD work was also framed more generally at the meso-level as directed towards enabling communities and social networks to ‘tend to their own dying’, which includes but surpasses individual and family care. At the macro-level there was significant discussion about the role of EOLDs in changing the culture of dying and increasing death literacy on a societal level through advocacy and education, including alignment with Compassionate Community initiatives.

**Figure 1.**
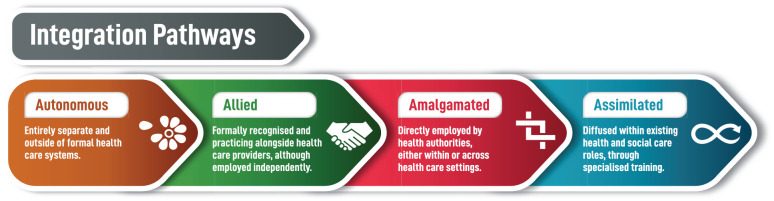
Potential EOLD integration pathways into existing health care systems.

In the context of future integration pathways, there were four main models discussed (see Figure 1). The most distal point is ‘autonomous’, entirely separate and outside of formal health systems. The second model is one where EOLDs are ‘allied’, formally recognized and practicing alongside current health care providers, although independently. The third possibility is an ‘amalgamated’ model, where EOLDs are directly employed by health authorities, either within or across specific care settings. Finally, the EOLD role could be fully ‘assimilated’ into existing care roles, as a form of specialist awareness and skills training for existing health and social care providers. For most participants, none of these models necessarily precluded the others.

In reflecting on the rapid growth of the field, symposium participants identified the need for better training and practitioner support. Some UK participants noted that EOLD UK has a mentorship requirement within its Code of Practice, as do some other national level organizations. However, participants also noted that in many instances training practicum requirements are often minimal, such as providing a small number of hospice volunteer hours, which may not include any significant contact with patients or families, be based on asking for a reflection about a personal experience or role-play with a friend or family member. Consequently, there was significant interest in developing stronger practicum and mentorship requirements within training programmes.

American participants noted that the United States has national practitioner networks such as the National End-of-Life Doula Alliance (NEDA), and their ‘EOL Doula Proficiency Badge’ which tests ‘core competencies’ was positively referenced several times. American participants also highlighted that EOLDs have representation within the National Hospice and Palliative Care Organization. Canadian participants also identified The EOLD Association of Canada as being central to the development of the field in that country. Participants generally – although not universally – felt these types of organizations and initiatives have strengthened the development of a collective field of practice.

Overall, participants were clear about the challenges being faced, and some held very strong opinions about how the EOLD role should or should not develop. At the same time, there was also significant reflexivity about the potential benefits of having space for divergent or ambivalent perspectives:

Lack of funding [for EOLDs] can bring about opportunities and lack of regulation can mean more flexibility to work; at the same time there is less or lower compensation. Institutional and community-led work at the end of life both have a place and role.

As the above quote indicates, discussion about the future pathways of the EOLD role often contained a strong general sense that the field needs to encourage and support a diversity of practice and practitioners, or in the words of one participant ‘the need for multiple tracks under one umbrella’.

Finally, participants identified that creating resilient developmental pathways would benefit from further ‘neutral’ discussion opportunities within a collaborative international context. Benefits of developing an ongoing international forum included establishing a more ‘robust international presence’ for what is becoming a transnational movement; enhanced understanding of integration initiatives from regions at the forefront of this work; learning about different health and social care structures and regulations; making the movement more inclusive beyond predominantly visible English-speaking white middle-class women from the global north; and in linking individual EOLD care services across international boundaries. The desire for cross-country collaboration was also discussed in relation to developing an international research agenda for the field. While a few participants noted an interest in better understanding of similar roles within different cultures, particularly the global south, this did not appear to be a main theme of the symposium.

## International similarities and differences

There have been few opportunities for EOLD practitioners to collectively discuss their work and interests within an international context. Analysis of conversations during the EOLD2022 Symposium – the first international symposium of its kind – evidenced many overlapping themes that have been detailed here. There were, however, also several differences of note between countries that require further consideration. The first is how best to describe those who use EOLD services. The nomenclature of ‘clients’, ‘patients’, ‘people we support’ or otherwise is of considerable importance as it reflects how EOLDs want their work to be categorized and situated (or not) within existing health systems. A second but related difference is between funding for health care systems, which has been noted elsewhere.^3^ Australia, Canada and the United Kingdom have publicly funded and administered health care systems that provide universal coverage based primarily on taxation, unlike the US’s mixed public/private system, whereas other countries may have primarily private health care. Consideration needs to be given to the unintended consequences of EOLDs integrating within mixed economies of care and diverse health systems that may be differentially shaped by profit-based motives.

Interestingly, while practitioner and legislative differences in after-death care have been noted by other researchers,^3^ this topic did not feature prominently in participants’ discussions during the symposium. In addition, reference to people performing similar roles in non-predominantly English-speaking countries was not a significant point of discussion. This may have been due to a focus on countries where the role is currently most developed, as other research with EOLDs has indicated a strong interest in cross-cultural comparative understandings of community-led end-of-life care.^3,4,7^

While regional differences were noted, participants primarily focussed on the similarities across represented countries and/or what they could learn from EOLDs in other countries. For example, there was significant interest in the two collaborator presenters (EC1 and JM) who provided examples of pilot projects integrating EOLDs and/or EOLD principles into regional health authorities. In another example, our Australian collaborator (AM) generated a great deal of interest about working with those who have requested assisted dying, and which has been legislated in parts of or all of the main countries represented, except the United Kingdom. However, UK participants were equally interested, in anticipation of legislation passing at some not-too-distant future point. A third example was the link Canadian and Australian participants made between their colonial histories in context of Indigenous end-of-life issues and initiatives. Some American participants also foregrounded the role of institutional (including biomedical) racism towards African Americans, Hispanic and Latino Americans, and other people of colour. Collectively, these perspectives generated the strong general sense that EOLDs understand death, dying and bereavement as social justice issues, and that the field of practice – as a whole – requires ongoing critical reflexivity as it develops and/or integrates within existing systems of care.

**Figure 2.**
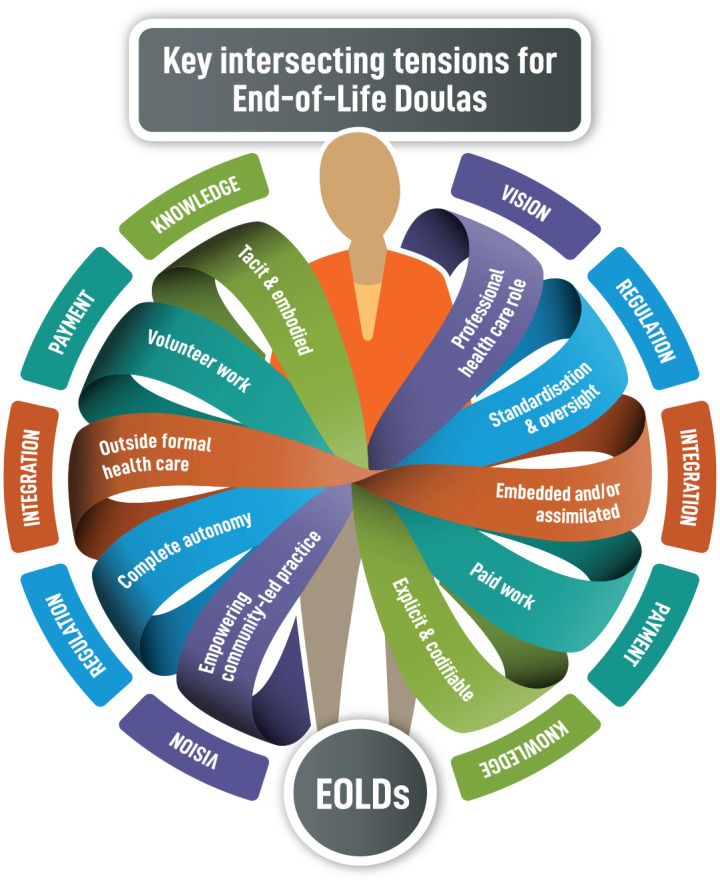
Key intersecting transnational tensions in the development of the EOLD role.

Perhaps most importantly, analysis of the symposium discussions found similarities in the key issues facing the development of EOLDs across international boundaries, and one of the central points of discussion across the 3 days was how best to negotiate these tensions. As the term ‘balancing’ was often invoked, these tensions are summarized and represented in Figure 2 as an intersecting spectrum (rather than merely as opposites) across the five domains, which participants most often referenced.

These tensions shaping the development of the EOLD role are embedded within broader socio-epidemiological changes that are reshaping the end of life and end-of-life care in global north countries more broadly. These changes include but are not limited to unprecedented rates of ageing, multimorbidity, frailty and dying; neo-liberal retrenchment of welfarist states resulting in increasing structural inequalities and vulnerabilities;^25^ desires to devolve end-of-life care to home or home-like settings;^26,27^ restructuring of family and community relations;^28^ growing popularity of the loosely-knit ‘death positivity’ movement;^29^ increasing states-level policy and public discourse about the need for active and responsible decision-making at the end of life, including advance care planning,^30^ and concerns about autonomy and suffering reflected in the growth of assisted dying legislation.^10^ Many of these issues were referenced – in some shape or form – by symposium participants in their discussions about the developments, disruptions, dilemmas and directions of the EOLD movement. Consequently, this emerging care role, the practice ambivalences identified and the reflexivity evidenced, makes EOLDs a critical and unique group through which to explore shifts in our collective yet ambivalent desires about how best to organize dying, death and bereavement in the 21st century within the global north.

## Conclusion

The International End-of-Life Doula (EOLD, 2022) Symposium was the inaugural international symposium or conference of its kind. It offered the world’s first opportunity to critically reflect on similarities and differences in the development of EOLDs across national boundaries. This article summarized the key concerns, issues and interests among practitioners and key stakeholders who were in direct conversation with each other across seven countries. This is the first event that has brought EOLDs together internationally, and our results indicate that there are fundamentally similar developmental issues across countries.

Our goal has been to critically examine and summarize current issues shaping the field and thereby enhance the resilience of EOLDs to provide innovative care and to act as meaningful cultural change agents within their diverse communities, as well as across international boundaries. Based on the symposium findings, we have developed an innovative framework of key intersecting practice tensions that are shaping this new care role. We believe this framework is relevant across both national and international contexts, and that it will be of foundational importance to a diversity of stakeholders interested in shaping the future development of this emerging transnational movement.

## Declarations

**Ethics approval and consent to participate:** Ethical approval for taking de-identified notes was granted by the University of Glasgow (approval id. 400210167). Symposium participants were provided with a plain language summary ensuring they were aware that notes would be taken throughout the event, and recorded informed consent was required before they were able to register

**Consent for publication:** Consent for publication was granted through recorded informed consent process.

**Availability of data and materials:** Data were not kept beyond the project due to the potential for personal identification.
